# A week-long meditation retreat decouples behavioral measures of the alerting and executive attention networks

**DOI:** 10.3389/fnhum.2014.00069

**Published:** 2014-02-17

**Authors:** James C. Elliott, B. Alan Wallace, Barry Giesbrecht

**Affiliations:** ^1^Department of Psychological and Brain Sciences and Institute for Collaborative Biotechnologies, University of California at Santa BarbaraSanta Barbara, CA, USA; ^2^Santa Barbara Institute for Consciousness StudiesSanta Barbara, CA, USA

**Keywords:** meditation, attention network test, orienting, alerting, shamatha meditation, visual attention, executive attention

## Abstract

Previous studies have examined the influence of meditation on three functionally different components of attention: executive control, alerting, and orienting. These studies have consistently found that meditation training improves both executive attention and alerting, but there has not been a consistent and clear effect of meditation training on orienting. In addition, while previous studies have shown that the functional *coupling* of the alerting and executive networks increases the processing of task irrelevant stimuli, it is unknown if participating in a meditation retreat can decouple these components of attention and lead to improved performance. The current study investigated the influence of a week-long intensive meditation retreat on three components of attention by randomly assigning participants to either pre- or postretreat testing groups. A modified attention network test (ANT) was used. Executive attention was measured as the difference in response time (RT) between congruent and incongruent task irrelevant flankers (conflict effect). Reflexive and volitional orienting were measured by manipulating cue validity and stimulus onset asynchrony (SOA). The coupling of executive attention and alerting was measured by examining flanker interference as a function of the SOA of an alerting cue. The meditation retreat improved task based indices of executive attention, but not reflexive or volitional orienting. There was clear behavioral evidence of coupling between executive attention and alerting in the preretreat group, as the conflict effect peaked when an alerting cue was presented 300 ms before the target. Importantly, there was no increase in the conflict effect for the postretreat group. This is consistent with the notion that the retreat decoupled the executive and alerting networks. These results suggest that previously reported improvements in the executive and alerting networks after meditation training might be mediated by the same underlying mechanism.

Identifying effective strategies for improving attention could have drastic impacts on everyday life: from saving lives by preventing car accidents to attenuating cognitive decline associated with aging. Within recent years, there has been growing interest in using meditation as a means to improve attention and related cognitive processes. Studies have reported encouraging evidence suggesting that meditation activates multiple attention-related neural networks and improves the functioning of executive attention (Brefczynski-Lewis et al., 2007; Jha et al., 2007; Tang et al., 2007; Kozasa et al., 2011), vigilance/alerting (Jha et al., 2007; MacLean et al., 2010), and visuospatial processing (Kozhevnikov et al., 2009).

These studies, and others in the literature, have used a variety of different meditation techniques. Importantly, those meditation techniques traditionally classified as shamatha (more recently referred to as focused attention; Lutz et al., 2008) explicitly encourage the development of focused and sustained attention (Wallace, 2006). During shamatha meditation, the practitioner can choose to attend to any object or sensation, such as the feeling of the breath at the nostrils, the face of a loved one, or religious iconography (Tsongkhapa, 2002; Wallace, 1998). However, the defining characteristic of a shamatha meditation is that the practitioner attempts to cultivate two related but distinct mental faculties: mindfulness and introspection (Wallace, 2006). Mindfulness, in the classical context, refers to the ability to maintain attention to a chosen object of meditation (e.g., the breath) over time. Introspection, on the other hand, refers to the ability to monitor the meditative process. For example, while one is meditating it is common that the mind will wander from the chosen object, which indicates a lapse in mindfulness. If the meditator is properly applying introspection by monitoring the quality of attention, the meditator will recognize that the mind has wandered off task and attempt to reengage with the chosen object of meditation. Finally, shamatha practice is often practiced immediately prior to or in conjunction with other types of meditation (Wallace, 2006; Jha et al., 2007; Lutz et al., 2008).

While it is clear that shamatha meditation emphasizes the cultivation of attention, it is important to recognize that attention is not a unitary phenomenon and consists of multiple cognitive and perceptual functions. For instance, William James suggested that these functions could be classified as sensorial or intellectual, voluntary or passive, and either immediate or derived (James, 1890). More recently, one neurocognitive framework of attention proposed by Posner and Petersen suggests that attention consists of at least three subsystems: alerting, orienting, and executive control (also see Posner and Petersen, 1990; Fan et al., 2002). While there are certainly several other frameworks that characterize attentional operations (e.g., Corbetta et al., 2008), the general framework proposed by Posner and Petersen has been the focus of several foundational studies that explored the influence of meditation on attention (Jha et al., 2007; Tang et al., 2007). According to this framework, the three subsystems of attention perform different functions and are anatomically distinct (Posner and Petersen, 1990; Petersen and Posner, 2012). The alerting network initiates and sustains a state in which the participant is prepared to respond to upcoming stimuli, both in response to a warning cue and over an extended period of time (Posner, 2008). The orienting network mediates the selection of relevant information and is often manipulated by cuing attention to a particular spatial location (Corbetta and Shulman, 2002). Finally, the executive network is involved in error monitoring and resolving perceptual, cognitive and response conflict (Fan et al., 2002; Petersen and Posner, 2012). Since the goal of shamatha meditation is to develop sustained attention (alerting network), and since a meditator must monitor the quality of attention (executive network) and reorient attention when the mind has wandered (orienting network), it seems reasonable to surmise that meditation could influence each of the three functions of attention.

Consistent with this descriptive analysis, one of the more reliable findings in the meditation literature is that training influences both behavioral and neural measures of the executive network. Tang et al. (2007) had college age volunteers participate in either an integrated mind body training (IBMT) or a relaxation training that consisted of an initial period of instruction followed by 20 min of meditation or relaxation practice for 5 days (totaling 100 min). The attention network test (ANT) was given to all participants prior to and immediately following the training conditions. The standard ANT is a laboratory task designed to measure efficiency scores for the executive, orienting, and alerting networks of attention (Fan et al., 2002). Specifically, on each trial of the ANT participants are presented with an arrow target that is surround by task-irrelevant arrows pointing in the same direction (congruent flankers) or different direction (incongruent flankers) than the central target. Furthermore, the target is preceded by one of three cue conditions: a spatial cue that is 100% valid, a spatially neutral double cue, a spatially neutral central cue, or no-cue. The efficiency of executive attention is defined as the response time (RT) difference between congruent and incongruent flanker conditions. Orienting efficiency is defined as the difference in RTs between the spatial cue and the central cue conditions. The alerting efficiency score is defined as the RT difference between the double cue and the no-cue condition. Tang et al. (2007) observed that the IBMT reduced the influence of task irrelevant flankers, suggesting that IBMT increased the efficiency of the executive control network for this college age sample. However, there was no influence of the meditation training on either the alerting or orienting efficiency scores. In line with the improvement in executive attention, neuroimaging evidence from participants engaged in an 11 h IBMT have shown that IBMT can increase the integrity and efficiency of connections, measured as an increase in fractional anisotropy using diffusion tensor imaging, to the anterior cingulate cortex (ACC), a structure commonly associated with executive attention (Tang et al., 2010). Furthermore, other studies have shown that meditators have decreased activity in the ACC during incongruent trials of a Stroop task, suggesting an increase in the efficiency of the executive attention network (Kozasa et al., 2011).

Unlike the executive attention network, the influence of meditation training on the orienting network is much less clear. A number of studies have investigated the influence of meditation training on orienting, as measured by the ANT or more typical orienting tasks, and the results from these studies have been mixed. For example, Jha et al. (2007) found a larger RT difference between neutral and spatial cue conditions (i.e., the ANT index that measures the orienting network) when meditators were compared to a control group, but another group found that experienced meditators showed a decrease in the RT difference between the neutral and spatial cue (van den Hurk et al., 2010). Hodgins and Adair (2010) used a more typical orienting task that was not embedded within the ANT and found that meditators had decreased RTs to invalid cues compared to a control group with no meditation experience. This suggests that meditators were quicker to reorient attention to an invalidly cued target. In contrast to these conflicting results, neither Tang et al. (2007) nor Baijal et al. (2011) observed any influence of meditation training on the orienting efficiency score measured by the ANT. Thus, the overall influence of meditation on the orienting network is inconsistent and unclear.

With respect to the alerting network, studies have shown an influence on alerting, which is also known as vigilance or sustained attention (Posner, 2008). Specifically, Jha et al. (2007) found that compared to a control group, a younger subgroup that completed a month long meditation retreat had smaller alerting scores. In the ANT task used by Jha et al. (2007) alerting was measured as the difference between double cue and no-cue trials. The reduction in the alerting score was driven by a decrease in reaction time to no-cue trials and suggests that the meditators were more vigilant. Baijal et al. (2011) found similar improvements in alerting in a shamatha meditation training for children. Finally, using a randomly assigned wait list control design, MacLean et al. (2010) found that intensive long-term meditation training both increased perceptual sensitivity and decreased the typical vigilance decrement. All of these findings suggest that meditation training can increase the efficiency of the alerting network.

## Present aims

The aforementioned studies clearly demonstrate that various types of meditation training can influence multiple aspects of attention. However, two fundamental questions about the nature of this influence remain. First, the influence of meditation on the orienting network is unclear. This could be because orienting has typically been measured using the standard ANT, which uses a 100% valid cue at a peripheral location and, as a result, the observed orienting effects represent the interaction between reflexive and volitional attention (Ristic and Kingstone, 2006). This seems particularly likely given that the only study to include the typical manipulation of cue validity found that meditators were quicker to reorient attention to an invalid cue (Hodgins and Adair, 2010). The ANT also does not use the standard manipulation of stimulus onset asynchrony (SOA) between the cue and target to assess the time course of attentional orienting (Müller and Rabbitt, 1989).

Finally, none of the studies reviewed (Jha et al., 2007; Tang et al., 2007; van den Hurk et al., 2010; Baijal et al., 2011), nor any other study on meditation and attention, have addressed whether meditation influences the functional segregation or coupling of the different functions of attention, which is one of the key questions that the ANT has been used to assess. Specifically, the initial study of the ANT concluded that the executive, alerting, and orienting networks are independent (Fan et al., 2002). Yet, there clearly could be interactions between the three aspects of attention that the original ANT was not able to assess (Fan et al., 2009; MacLeod et al., 2010). For instance, by including manipulations of cue-target SOA, more recent studies have confirmed that functional coupling between the executive and alerting networks increases the conflict effect at SOAs of around 400 ms, but this effect is not observed at later SOAs (Fan et al., 2009; Weinbach and Henik, 2013). This coupling is thought to reflect a competition for neural resources shared by these two networks (Fan et al., 2009). Critically, while previous studies have found that meditation training influences alerting and executive attention, it is unknown if meditation training decouples these two attention networks, which would decrease the influence of task irrelevant information.

In order to address these questions in the present study, participants that registered for a week-long meditation retreat, which included 3–4 h of shamatha meditation per day, were randomly assigned to a pre- or postretreat group. Like a matched comparison group (e.g., Lutz et al., 2004; Slagter et al., 2007; Zanesco et al., 2013) and a wait list control group (MacLean et al., 2010), this design does not isolate the specific influence of meditation as compared to the entire meditation retreat, which includes, among other things, the following potential influences: the physical and social environment, the lectures and interactions with the instructor, and the lack of outside obligations (for a review of these issues, see Davidson, 2010; Slagter et al., 2011). However, compared to using a matched comparison group, the current cross-sectional design with random assignment to pre-or postretreat group effectively controls for issues commonly associated with the cohort effect in cross-sectional designs and individual differences in between subjects designs.

As mentioned, one possible explanation for the inconsistency in the previous studies examining the influence of meditation on orienting is that the standard ANT combines both reflexive and volitional cues and does not include a SOA manipulation. In order to determine if this contributed to the inconsistency, each participant in the current experiment completed two versions of the ANT, one that used nonpredictive peripheral (i.e., reflexive) cues and one that used central predictive and symbolic (i.e., volitional) cues. Additionally, the standard ANT uses a fixed 500 ms cue-target SOA (Fan et al., 2002). In the current experiment cue-target SOAs of 100, 300, and 600 ms were included in order to explore both the influences of reflexive and volitional cues on RT as a function of SOA and the relationship between the alerting and executive networks. In line with previous research on meditation, we predicted that a meditation retreat of this duration and intensity would increase the efficiency of the executive network of attention. Given the ambiguity in the literature with regards to the orienting network, we had no clear predictions about the influence of a meditation retreat on the orienting component. Furthermore, based on studies that have shown that meditation also influences the alerting network, we predicted that participation in the meditation retreat would decouple the alerting and the executive networks of attention.

## Methods

### Participants

Fifty-four volunteers recruited from retreats held in Santa Barbara, California, completed the entire experiment. Participants were recruited for the study after registering and paying for the retreat, which ranged from $600 to $1095 depending on the accommodations the participant selected. The UCSB Human Subjects Committee approved all procedures and informed written consent was obtained from all participants.

### Meditation retreats

Participants were recruited from three different 7 day retreats that were taught by one of the authors (B.A.W.) and were organized by the Santa Barbara Institute for Consciousness Studies. The retreats consisted of lectures, discussions, and meditations. Participants arrived on day 1, which included an evening introduction and guided meditation. Days 2–6 consisted of lectures, discussion, and 3–4 h of meditation that was done in a group setting. On the 7th day, participants met for the morning session and the retreat ended at noon. Participants were given explicit instructions both before and during guided meditations at the beginning of the retreat, though the frequency of instruction during the guided meditations decreased as the retreat continued. Across all three retreats the sessions consisted of shamatha meditations, including mindfulness of breathing and meditations on the Four Immeasurables (e.g., loving kindness; see Wallace, 2006). Importantly, these are all shamatha meditations that emphasize physical and mental relaxation and maintaining attention on the object of meditation (Wallace, 2006). During all of the meditations, participants were instructed to focus attention on a specific meditation object (e.g., the breath or a particular individual). Furthermore, during the meditations on the Four Immeasurables participants were also instructed to practice cultivating a particular affective stance (loving kindness, compassion, empathetic joy, or equanimity) towards the object of meditation

Some of the participants stayed on site at the Santa Barbara Mission, whereas others slept at home and commuted to the retreat location daily. The retreats where “semisilent”, such that participants were encouraged to maintain silence during the retreat, but were not obliged to do so.

### Attention network test

**Figure 1:**
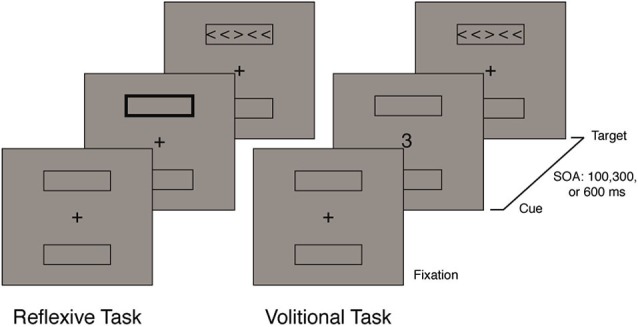
**Schematic representation of both the reflexive and volitional ANT**.

Two variations of the ANT (Fan et al., 2002) were created, one with volitional cues (Kasper et al., 2012) and one with reflexive cues. During the volitional orienting task, the numbers 3 and 9 were spatial cues that indicated the likely target location. Each number cued either the upper or lower target location (see Figure 1), and this spatial cue was valid on 80% of the trials. Participants were informed about the validity of the cue and instructed to use this information. The mapping of cue identity to target location was counterbalanced across subjects.^1^ There was also a neutral cue (0), which did not provide the participants with any spatial information.

During the reflexive orienting task, the uninformative spatial cue consisted of an increase in thickness of one of the two rectangles surrounding the possible target locations. Because this cue occurred in the target location on 50% of the trials and in the nontarget location on 50% of the trials, this cue did not provide the subject with any information about the upcoming location of the target. Furthermore, in the reflexive task, the spatially neutral cue consisted of an increase in thickness of both the rectangles surrounding the possible target locations. Figure 1 shows the sequence and layout of each of the tasks.

In both the reflexive and volitional task, the SOA between the cue and target was manipulated, which is typically used in studies looking at both attentional orienting (Posner, 1980; Müller and Rabbitt, 1989) and alerting (Fan et al., 2009). Overall, this modified ANT can assess executive attention as the difference between the congruent and incongruent conditions. Furthermore, both reflexive and volitional orienting can be independently assessed by examining the interaction between cue (valid/invalid) and SOA in each task. Finally, the functional coupling of the alerting and executive attention networks can be assessed by examining the interaction between congruency and SOA in the neutral alerting cue conditions of each task.

Regardless of cue type, the tasks had a similar trial structure. Participants initiated each block of trials by pressing the space bar. Each trial started with the presentation of two rectangles (2.2^°^ × 0.52^°^) that were onscreen throughout the task centered at 0.65^°^ above and below fixation and a fixation cross that was on the screen for a random duration of 400–1600 ms (drawn from a uniform distribution and rounded to a multiple of the frame rate of the monitor). A cue, either reflexive or volitional, was then presented for 100 ms. Following a SOA of either 100, 300, or 600 ms, a string of five arrows appeared in either the upper or lower rectangle. The task was to indicate the direction of the middle arrow while ignoring the flanking arrows that could be either congruent (>>>>>) or incongruent (>><>>). Each stimulus (i.e., target and flankers) was 2.08^°^ × 0.52^°^. Participants were instructed to respond as quickly and as accurately as possible by pressing one of the two corresponding keys on the keyboard. The target display remained on the screen until a response was made or for a maximum of 1700 ms. This was followed by the fixation cross and target location markers for 400 ms and the start of the subsequent trial. Participants sat 110 cm from a 19″ CRT monitor. They were instructed to keep their eyes fixated at a central crosshair throughout the duration of the task, however this was not verified following initial instruction.

### Questionnaire

At the end of the retreat participants were given a questionnaire. This questionnaire assessed the participants’ previous meditation experience, clinical or neurological conditions, use of psychoactive medicines, and retreat session attendance throughout the week. Questions were also asked about the extent to which they enjoyed the retreat and whether or not the experiment intruded in their retreat experience.

### Design

At the beginning of the retreat, participants were randomly assigned to either the pre- or the postretreat group. This cross-sectional design with random assignment was chosen in order to maximize the time in a single session, which is required to isolate the different attention networks, while also minimizing the time that the experiment required of the participants. This was particularly important given that retreat participants paid to attend the week-long retreat and were not compensated for their participation in the study. All participants started the retreat on day 1. The preretreat group participated in the computer task on either day 1 or 2 of the retreat and the postretreat group participated on either day 5 or 6 of the retreat. There were no experimental sessions on the 7th day of the retreat because the retreat ended at noon. Hence, the key between subjects factor was time (pre or post). The within subjects factors for each attention task were cue type (valid, invalid, and neutral), SOA (100, 300, and 600 ms) and target type (congruent and incongruent). The order of the volitional and the reflexive tasks were counterbalanced across subjects.

## Results

Seven participants reported that they had been diagnosed with either clinical or neurological disorders (e.g., depression, bipolar disorder, traumatic brain injury, recent concussion) and an additional six participants reported that they were taking psychoactive medications at the time of the retreat. These participants were excluded from all analyses. After excluding these participants, there were a total of 41 participants included in the analyses. There were 22 participants in the pregroup and 19 participants in the postgroup. Of those assigned to the pregroup, 7 participated on day 1 and 15 on day 2.^2^ For the postgroup, 12 individuals participated on day 5 and 7 individuals on day 6. Two-tailed *t*-tests revealed no differences between the groups in age (*t*_(39)_ = 0.307, *p* = 0.76; mean (SEM): pre = 52.54 years (2.5), post = 53.8 years (3.2)) daily meditation practice (*t*_(38)_ = 0.22, *p* = 0.82; pre = 46.5 min (10.4), post = 56.5 min (12.7)), or total number of days spent in retreat during the last 10 years (*t*_(38)_ = 0.315, *p* = 0.54; pre = 44.3 days (10.3), post = 40.7 days (11.9)). One participant omitted a response for the daily meditation practice question and a second participant omitted a response for the question concerning the number of days spent in retreat during the last 10 years. Therefore, these participants were excluded from tests comparing the groups on those measures. In order to explore individual differences due to each of these measures, analyses were also conducted with the daily meditation and the days in retreat measures separately included as covariates in a repeated measures ANOVA. Neither of these measures were significantly related to any of the key performance measures. Finally, all subjects in both groups reported attending all scheduled sessions of the retreat. Therefore, there was no recorded variance in the meditation time across participants within each retreat. However, small, undocumented modifications to the duration and number of meditation sessions were occasionally made to accommodate the teaching goals of each individual retreat.

The analysis was done in three parts. First, we examined the influence of the retreat on the executive network of attention. We next investigated the influences of meditation on reflexive and volitional orienting. Then we examined the extent to which the meditation retreats influenced the interaction between the executive and alerting networks of attention.

### Executive attention

**Figure 2:**
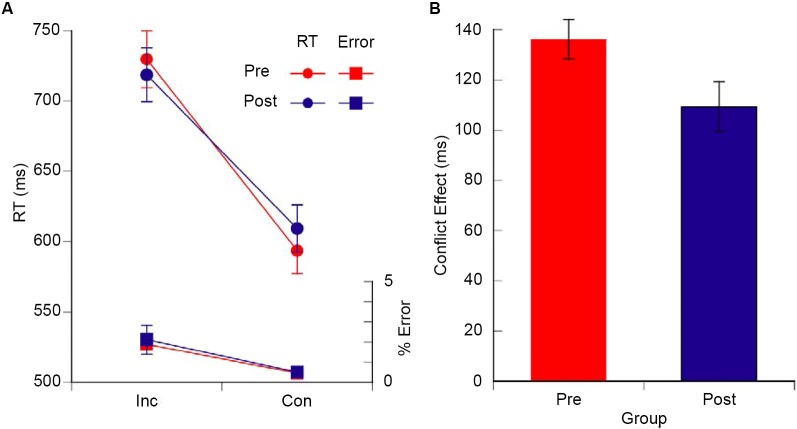
**(A)** Mean RT (ms), error rate, and **(B)** conflict effect (RT incongruent—RT congruent targets) for the pre (red) and the post (blue) retreat groups. Error bars are S.E.M. in all figures.

The magnitude of the flanker congruency effect (i.e., conflict effect) was calculated in order to examine the influence of meditation training on the executive network of attention. In line with previous research using ANT type tasks (Fan et al., 2002), the conflict effect for each individual was computed as the mean RT (or error) of all incongruent trials minus the mean RT (or error) of all the congruent trails. The mean RTs and error rates are shown in Figure 2A and the RT conflict scores are shown in Figure 2B. The RT conflict effect for the postretreat group was smaller than the preretreat group, *t*_(39)_ = 2.15, *p* = 0.018 (one-tailed), Cohen’s *d* = 0.688 (see Figure 2). There was no significant difference between the pre- and postgroup for the error conflict effect, *t*_(39)_ = 0.323, *p* = 0.749.

### Volitional orienting

**Figure 3:**
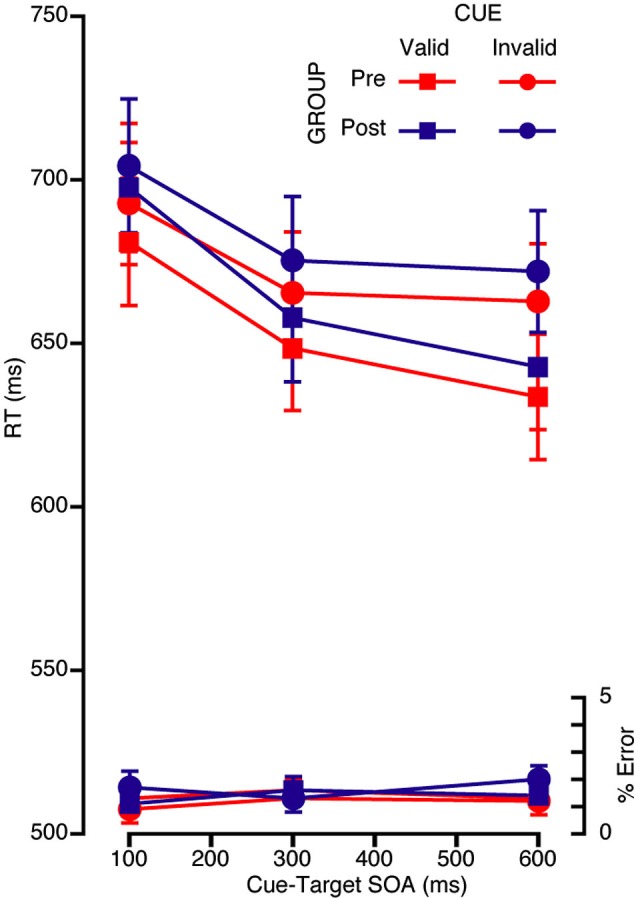
**Mean RT (ms) and error rate for each SOA and validity condition for the volitional attention task for the pre (red) and the post (blue) retreat groups**.

The mean RTs (accurate trials only) and error rates were submitted to a repeated measures ANOVA with three within subjects factors of cue (valid or invalid), congruency (congruent or incongruent), and cue-target SOA (100, 300, or 600 ms) and one between subjects factor (pre- or postretreat group). The analysis of the RT data revealed a two-way interaction between cue and SOA, *F*_(2,78)_ = 10.28, *p* < 0.001, such that the difference between valid and invalid cues increased at the longer SOAs (see Figure 3). No interactions with group approached significance in either the RT or error data.

### Reflexive orienting

**Figure 4:**
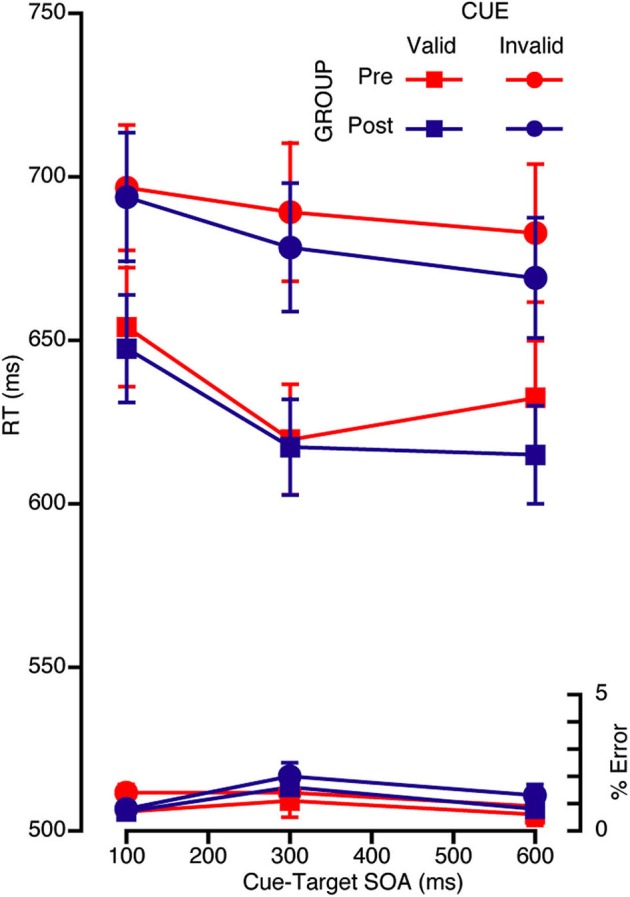
**Mean RT (ms) and error rate for each SOA and validity condition for the reflexive attention task for both the pre (red) and the post (blue) retreat groups**.

As with volitional orienting, the mean RTs and error rates from the reflexive task were analyzed with a repeated measures ANOVA with three within subjects factors of cue, congruency, and SOA and one between subjects factor of group. Other than an interaction between group and congruency in RT (*F*_(1,39)_ = 4.92, *p* = 0.032), there were no main effects or interactions with group in either the error or the RT data. With respect to cue validity, there was a two-way interaction between cue and SOA, *F*_(2,78)_ = 5.96, *p* = 0.004, such that RT decreased with SOA and RT to validly cued targets was faster at 300 ms compared to the other two SOAs (see Figure 4).^3^

### Alerting and executive network interactions

**Figure 5:**
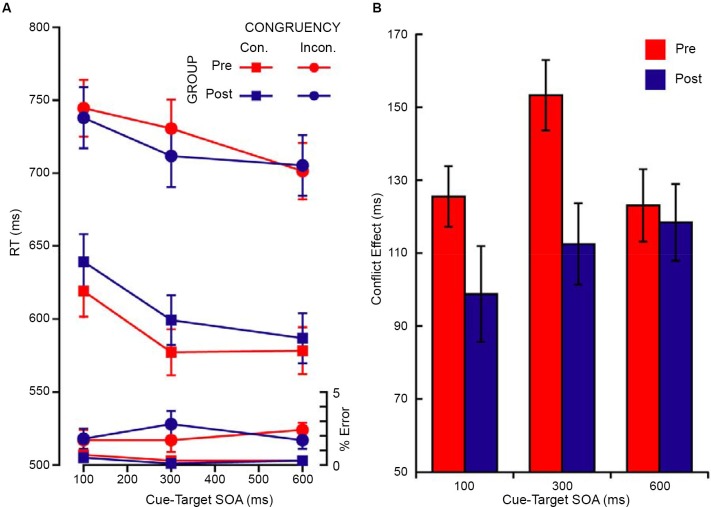
**(A)** Mean RT (ms), error rate, and **(B)** conflict effect (RT incongruent—RT congruent targets) in the neutral cue condition as a function of the cue-target SOA for both the pre (red) and post (blue) retreat group.

In order to examine the influence of the meditation retreat on the functional coupling between the executive and alerting networks, the mean error and RT data (see Figure 5A) from the neutral cue for each of the two tasks was submitted to a repeated measures ANOVA with three within subjects factors of task, congruency, and SOA and one between subjects factor of group. For the preretreat group, the cue influenced the congruency effect in a manner consistent with previously reported findings, such that there was an increase in the difference between congruent and incongruent targets at the 300 ms SOA compared to the 100 and 600 ms SOAs (see Figure 5B). However, this specific influence of the neutral cue was not observed for the postretreat group. This pattern was confirmed by a three way interaction between congruency, SOA, and group (*F*_(2,78)_ = 5.18, *p* = 0.008, $\eta_{p}^{2}=0.117$) which was driven by the difference at 300 ms SOA (*t*_(39)_ = 2.78, *p* = 0.008).

## Discussion

The present experiment investigated two questions: (1) does a meditation retreat improve the efficiency of the orienting network when volitional and reflexive attention are independently assessed; and (2) what is the influence of a meditation retreat on the functional coupling of the executive and alerting attention networks. The results of the current experiment show that an intensive week-long meditation retreat can improve the efficiency of executive attention and decouple the alerting and executive attention networks.

### Meditation and the orienting network

Despite the influence of the meditation retreats on executive attention in the current study, there was no observed effect on either reflexive or volitional orienting. While this finding is consistent with two previous studies that reported no effect of meditation on attentional orienting (Tang et al., 2007; Baijal et al., 2011), it is inconsistent with one paper that reported a larger orienting effect (Jha et al., 2007) and two other studies that reported a smaller orienting effect (Hodgins and Adair, 2010; van den Hurk et al., 2010). There are at least four different, though not mutually exclusive, explanations for this discrepancy in the literature: (1) the orienting tasks used have not been sensitive enough to detect a consistent difference due to meditation; (2) meditation requires orienting, but not to the extent required to observe robust behavioral improvements measured with laboratory tasks; (3) the orienting network is not utilized during meditation; or (4) the variability reported in the literature is due to differences in meditation training (e.g., meditation style or duration).

In line with the first option, MacLeod et al. (2010) have shown that the ANT orienting and alerting efficiency scores are less reliable than the executive network score. However, they also note that the orienting score has the greatest power when using between subjects designs, even though this is not consistent with previous results (MacLeod et al., 2010). Another issue that could influence the sensitivity is that the ANT orienting cue is a combined reflexive and volitional cue. However, the current study included independent reflexive and volitional tasks and there was no difference between groups. This suggests that the inconsistency in the literature is not likely due to the combined reflexive and volitional cues used in previous versions of the ANT, though it is always problematic to interpret a null effect. As for the second possible explanation, shamatha meditation involves focusing on an object for an extended period of time. This is commonly interpreted as requiring the meditator to focus on the object, to recognize when the mind has wandered, and then to reorient attention to the initially chosen object. Given that the meditation object is typically a familiar stimulus already within one’s awareness, after one has recognized that the mind has wandered, the process of returning to the object of meditation might be too simple to improve the functioning of the orienting network. With regards to the third option, the process of returning to the object of meditation might be an error monitoring process (i.e., reactivating relevant goals), which would not involve the orienting network of attention. Neither option two nor three would predict an improvement in the efficiency of the orienting network. The fourth option also seems plausible, particularly since there is no formal way of classifying different styles of meditation. The best approach to determine how different meditations influence orienting is to experimentally manipulate meditation style within an experiment. Kozhevnikov et al. (2009) used this technique to explore differences between Deity Yoga and Open Presence meditation and found that Deity Yoga increased access to visuospatial processing resources compared to the Open Presence meditation. However, no experiments have yet to measure the influence of two different experimentally manipulated meditations on the orienting network or any other attention network.

### Functional decoupling of the alerting and executive networks

Results from the ANT have been used to argue that the attention networks are functionally independent (Fan et al., 2002). However, this independence has not been absolute. Specifically, in an ANT task that included cue-target SOAs of 0, 400, and 800 ms, Fan et al. (2009) found that there was an increase in the RT difference between congruent and incongruent trials when the targets were preceded by a double cue. These authors suggested that this pattern of results represented a functional coupling between the alerting and executive networks caused by both networks competing for access to shared neural resources, including the ACC (Fan et al., 2007). Alternatively, the functional coupling could be facilitated by modifications of one of the two networks. For instance, the alerting network typically increases stimulus processing, whereas the executive network resolves perceptual conflict (Fan et al., 2002, 2007). Thus, if the alerting network increases stimulus processing independent of task relevance, this would increase the amount of task irrelevant information that must be processed by the executive system and increase the difference between the congruent and incongruent RTs.

In the present study, we found that a week-long meditation retreat decreased the influence that an alerting cue has on the conflict effect at the 300 ms SOA. The current results are consistent with either of the two above interpretations. That is, the retreat could have improved the efficiency of the shared neural resource, such as the ACC, thereby allowing both the alerting and executive networks to operate in parallel. It is also possible that, without sharing resources, an increase in the capacity of executive attention mitigated the influence of the task-irrelevant stimulus processing induced by the alerting cue. On the other hand, the observed interaction could mean that the retreat decreased the extent to which the alerting cue facilitates unselective processing of upcoming stimuli, which would decrease the amount of task irrelevant information that must be processed by the executive network. Either way, our findings support the notion that meditation retreats decouple the alerting and executive attention networks. This raises the intriguing possibility that previously reported effects of meditation on alerting (Jha et al., 2007; MacLean et al., 2010; Baijal et al., 2011) and executive attention (Jha et al., 2007; Tang et al., 2010; Baijal et al., 2011) may be mediated by the same underlying improvement in the efficiency of a shared neural resource, possibly the ACC (Fan et al., 2007).

### Caveats and limitations

The interpretations of the present results are constrained by at least two possible issues. First, the current experiment cannot determine if meditation retreats have any long-term effect because participants were unavailable for follow up testing after the retreat concluded. This leaves open the possibility that any influence of the meditation retreat might only have lasted as long as the retreat itself. Second, as mentioned previously, the design of the current experiment cannot isolate the exact influence of the meditation practice from the overall experience of the retreat. Therefore, any effect observed in the current study could possibly be attributed to an aspect of the meditation retreat other than meditation, such as the lectures, group discussions, social interactions, and the general environment of the retreat. However, unlike standard cross-sectional designs, the potential confounds associated with the cohort effect were eliminated by randomly assigning participants to either the pre- or postretreat group.

## Conclusion

The current results show that a week-long meditation retreat can improve executive attention. However, the meditation retreat did not result in any observable improvement in either reflexive or volitional orienting, and the influence of meditation on the orienting network remains, at best, equivocal. Furthermore, the meditation retreat decreased the detriment associated with the functional coupling between the alerting and executive attention networks, suggesting that the performance of these networks was improved by the meditation retreat.

## Conflict of interest statement

The authors declare that the research was conducted in the absence of any commercial or financial relationships that could be construed as a potential conflict of interest.

## Footnotes

^1^(Ristic et al. 2006) have shown that the numbers 12 and 6 reflexively cue attention when targets are located above and below fixation, as these numbers are associated with these locations on a clock face. Using the numbers 3 and 9 should prevent any reflexive number cueing effects because these numbers would be associated with the left and right target locations.

^2^One possible explanation for any result with this design is that individuals that participated on day 1 were experiencing fatigue due to traveling or other factors related to arriving on site. In order to rule this out, analyses were also done that excluded those individuals tested on day 1, and the significance and pattern of the results remained the same.

^3^A follow up analysis was conducted including both volitional and reflexive orienting as factors in order to determine if this would be more sensitive to a difference between the pre and postretreat groups. Consistent with the conflict effect reported, there was a significant interaction between group and congruency, *F*_(1,39)_ = 5.174, *p* = 0.029. However, there were no significant interactions with group that included task, cue, or SOA.
